# Virome Analysis of Small Mammals from the Brazilian Amazon

**DOI:** 10.3390/v17091251

**Published:** 2025-09-16

**Authors:** Leonardo Henrique Almeida Hernández, Fábio Silva da Silva, Thito Yan Bezerra da Paz, Daniel Damous Dias, Bruno de Cássio Veloso de Barros, Bruno Tardelli Diniz Nunes, Lívia Medeiros Neves Casseb, Sandro Patroca da Silva, Pedro Fernando da Costa Vasconcelos, Ana Cecília Ribeiro Cruz

**Affiliations:** 1 Graduate Program in Virology, Evandro Chagas Institute, Health and Environment Surveillance Secretariat, Ministry of Health, Ananindeua 67030-000, PA, Brazil; leohenrique96@hotmail.com (L.H.A.H.); liviacasseb@iec.gov.br (L.M.N.C.); pedro.vasconcelos@uepa.br (P.F.d.C.V.); 2 Department of Arbovirology and Hemorrhagic Fevers, Evandro Chagas Institute, Health and Environment Surveillance Secretariat, Ministry of Health, Ananindeua 67030-000, PA, Brazil; fabiosilva.analises@gmail.com (F.S.d.S.); thitodapaz000@gmail.com (T.Y.B.d.P.); damous1994@gmail.com (D.D.D.); brunonunes@iec.gov.br (B.T.D.N.); spatroca@gmail.com (S.P.d.S.); 3 Graduate Program in Parasite Biology in the Amazon Region, Center for Biological and Health Sciences, Pará State University, Belém 66087-670, PA, Brazil; 4 Faculdade Vale dos Carajás, Parauapebas 68515-000, PA, Brazil; brunocvb@yahoo.com.br

**Keywords:** Chiroptera, deforestation, Didelphimorphia, metagenomics, Rodentia

## Abstract

The municipalities of Peixe-Boi and Santa Bárbara do Pará, both in the Pará State (eastern Amazon), have more than half of their territory deforested. Understanding the viral diversity in wildlife that inhabits the surroundings of human communities contributes to strengthening surveillance. Samples from eleven bats, seven opossums, and eight rodents from the two locations were screened by high-throughput sequencing for virome analysis. Viral reads were assigned into twenty viral families, from which the most abundant was *Retroviridae*. Host order, tissue type, and season showed a significant effect on viral composition. Five viral genomes of bat ERVs with intact genes were recovered, showing the need to understand their endogenous nature. In addition, a new Buritiense virus (*Hantaviridae*) strain was also obtained, supporting its circulation in Santa Bárbara do Pará and expanding its genomic information. Together, these findings reinforce the need for continuous surveillance in wild animals, especially in the Amazon region, to anticipate potential threats to public health.

## 1. Introduction

Zoonotic diseases are those transmitted from vertebrate animals to humans or from humans to other vertebrates. In addition to their impact on human health, zoonoses directly affect the economy, especially the livestock industry. Their effects can also be observed on ecosystems and the biodiversity of wild animals [1]. It is estimated that 60.3% of emerging infectious diseases described between 1940 and 2004 were zoonotic infections. More than two-thirds of these infections originated in wild animals, highlighting the deleterious effect of deforestation and climate change on wildlife, and for extension, on human health over the last few decades [2].

In the Amazon region, intense human activity driven by economic exploitation has resulted in a high rate of deforestation, reaching almost 40% [3]. Furthermore, the latest information provided by the Brazilian National Institute for Space Research (INPE) indicates that during 2024, the devastated area was 6288 km^2^, a decrease of 30.62% compared to the previous year [4].

The state of Pará stands out with the highest rate of deforestation within the Legal Amazon, which comprises the seven states of the Brazilian North region, as well as the northern part of the state of Mato Grosso and the northwestern part of the state of Maranhão, corresponding to 58.9% of Brazil’s territory. From 1988 to 2024, the state was responsible for the deforestation of an area of 172,435 km^2^, corresponding to 34.68% of all deforestation in the Legal Amazon during this period [4]. By 2024, the municipality of Peixe-Boi, located in the Northeast region of Pará and covering an area of 450.224 km^2^, had approximately 81.16% (365.43 km^2^) of its territory deforested. On the other hand, the municipality of Santa Bárbara do Pará is in the metropolitan area of Belém (capital of Pará state) and covers an area of 278.154 km^2^, of which 59.07% (164.31 km^2^) had already been deforested by the same year [4].

Progressive deforestation followed by urban and economic development increases contact between humans and the remaining wild animal populations in degraded areas. In this context, infections with zoonotic potential may find favorable conditions to emerge in humans [5]. That is the case with viruses, especially RNA viruses, which are the second-largest group of zoonotic agents that have emerged in human populations in recent years, because of their high mutation rates and easy adaptation to new hosts, a phenomenon known as spillover [2,6].

Chiroptera comprises the second largest order of mammals and have been shown to be important reservoirs and disseminators of zoonotic viruses due to factors such as their social behavior, ability to travel long distances, and even echolocation, given that the emission of ultrasonic waves generates aerosols that may be contaminated [7,8]. Rodents are also described as important virus reservoirs and, like bats, do not usually show clinical signs of disease, which contributes to the spread of pathogens [9,10].

The presence of these small mammals, as well as opossums, impacted by human activity in their original habitat, is becoming increasingly frequent in rural and peri-domicile areas, where they move to find food and shelter. This phenomenon promotes direct or indirect contact between humans and these animals, creating favorable conditions for the emergence of viruses that have already been described and those that have not yet been discovered [7,8,9,10].

The collection and analysis of samples from these animals is essential for conducting eco-epidemiological studies and comprises the One Health approach, since understanding the diversity of viruses circulating in these animal populations that inhabit the surroundings of human communities contributes to strengthening surveillance [1]. From this perspective, metagenomic analysis is a valuable tool for virus surveillance, capable of scanning the viral diversity represented in each biological sample by sequencing its genetic material. It has been used as an ally in the epidemiological surveillance of pathogens and decision-making in public health [11,12]. Hence, we applied the metagenomics analysis to assess the virus diversity in bats, opossums and rodents from Peixe-Boi and Santa Bárbara do Pará municipalities.

## 2. Materials and Methods

### 2.1. Sample Collection

**Figure 1:**
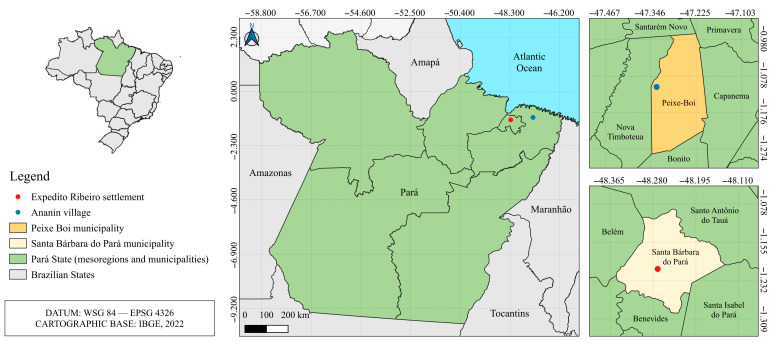
Sample collection location.

Biological samples from bats, opossums, and rodents were collected during an eco-epidemiological investigation in the Ananin village (1.10706 S, 47.33164 W), in the municipality of Peixe-Boi (Figure 1) in September 2015 and May 2016 and in the Expedito Ribeiro settlement (1.20426 S, 48.27002 W), in the Santa Bárbara do Pará municipality (Figure 1) in October 2014 and April 2015. This study is part of a major virus surveillance project authorized by the Ethics Committee on Animal Use of the Evandro Chagas Institute (IEC) under certificates numbers 21/2014, 25/2022 and 10/2024.

Animals were collected in the forest area and in the surroundings of human habitations using mist nets for bats and Tomahawk and Sherman traps for opossums and rodents. From the two expeditions in the Peixe-Boi municipality, twelve mammals were collected: seven bats, four opossums, and one rodent. The two expeditions in the Santa Bárbara do Pará municipality resulted in the collection of fourteen mammals: four bats, three opossums, and seven rodents. All were identified morphologically and were characterized in terms of length, weight, sex, and age.

The 26 collected animals were anesthetized with Zoletil^®^ 50 via an intramuscular route, followed by euthanasia through an overdose of lidocaine administered into the foramen magnum. The animals were necropsied, and the harvested viscera were divided into two samples for each animal: one liver sample and a pooled viscera sample with fragments of the spleen, lymph nodes, heart, and lungs. For bats, a brain sample was also collected. All 63 samples were stored in liquid nitrogen and transported to the Department of Arbovirology and Hemorrhagic Fevers of the IEC (Ananindeua, Brazil) and maintained at −80 °C until downstream processing.

### 2.2. RNA Extraction and cDNA Synthesis

Based on morphological identification, samples from animals from the same species and expedition were organized into pools containing a 5 mg fragment from each sample. From these pool samples and from individual samples of other animals, a total of 5 mg of tissue was homogenized in a microtube with 1 mL of TRIzol^®^ Plus reagent (Invitrogen, Waltham, MA, USA) and one 5 mm tungsten bead via the TissueLyser II system (Qiagen, Hilden, Germany) for 2 min at 25 Hz. RNA extraction was conducted using the PureLink^®^ RNA Mini Kit (Invitrogen) following the manufacturer’s protocol. The first and second strands of complementary DNA (cDNA) were synthesized by the SuperScript™ IV VILO™ MasterMix (Invitrogen) and the Second Strand cDNA Synthesis Kit (Invitrogen), respectively.

### 2.3. Library Preparation and Sequencing

The cDNA library for shotgun sequencing was prepared using the Nextera XT DNA Library Preparation Kit (Illumina, San Diego, CA, USA). Library quantification was performed using a Qubit 2.0 fluorometer (Invitrogen), and the fragmentation level assessment was evaluated with a 2100 Bioanalyzer instrument (Agilent Technologies, Santa Clara, CA, USA). Sequencing was performed on the NextSeq 500 System (Illumina) with the NextSeq 500/550 High Output Kit v2.5 (300 cycles—Illumina), using the 150 bp paired-end methodology.

### 2.4. Bioinformatic Analysis

#### 2.4.1. Data Filtering and Quality Control

Raw data were initially subjected to quality assessment using Fastp v.0.23.4 [13], configured to remove adapter sequences, reads shorter than 50 nt and reads with a base Phred quality score < 20. The removal of ribosomal reads was performed using SortMeRNA v.2.1 [14] based on the default parameters and default database provided by the software.

#### 2.4.2. Assembly and Alignment

The remaining reads were de novo assembled using MEGAHIT v.1.2.9 [15] (k-mers 21, 31, 41, 51, 61, 71, 81, 91 and 99) and SPAdes v.4.0.0 [16] (k-mers 21, 33, 55, 77). The obtained contigs from both assemblers were compared against the non-redundant protein (nr) and the RefSeq databases, both from NCBI, through DIAMOND v.2.1.9 [17] using Blastx.

#### 2.4.3. Viral Diversity Analysis

For taxonomic level, viral operational taxonomic units (vOTUs) were defined based on contigs from the dual alignment with a minimum bit-score of 80, an alignment identity threshold of ≥50% for viral family-level assignments, and an e-value restricted to 1^−10^. Taxonomic classification was performed using the Lowest Common Ancestor (LCA) algorithm in MEGAN v6.21.1 [18]. The resulting vOTUs were exported from MEGAN and mapped against the filtered reads using Bowtie2 v.2.5.4 [19] in default mode.

The read counts for each viral family were normalized by reads per million mapped reads (rpm) and log scale. Then, they were used to generate the heatmap and calculate the general metrics of alpha diversity (abundance, richness, and Shannon index) and beta diversity (principal coordinate analysis—PCoA), using the R language [20] with the pheatmap [21], vegan [22], and ggplot2 [23] libraries.

For alpha diversity, the Shapiro–Wilk statistical test was performed to assess normality and the Mann–Whitney or the Kruskal–Wallis test followed by Dunn’s test and Bonferroni correction for non-parametric distribution, considering a *p*-value < 0.05 as significant. For beta diversity, the PERMANOVA (Permutation Multivariate Analysis of Variance) test was performed based on 999 permutations, considering a *p*-value < 0.05 as significant.

#### 2.4.4. Inspection and Phylogenetic Analysis

Viral contigs previously examined using MEGAN were exported to Geneious Prime 2025.1.3 [24], where inspection, map-to-reference alignment (Geneious Mapper), raw data alignment against contigs and multiple sequence alignment (Clustal Omega v1.2.3) [25] were conducted.

Analysis of the identified viral genomes was conducted using their obtained sequences along with closely related sequences available in the GenBank (NCBI) database. Phylogenetic signal estimation and phylogenetic tree reconstructions based on the Maximum Likelihood method with bootstrap values set for 1000 replications were performed by IQ-TREE v.3.0.1 [26], which also defined the best substitution model. Tree visualization and editing were performed, respectively, via FigTree v.1.4.4 [27], which employs the midpoint rooting methodology, and Inkscape v.1.3.2 [28].

#### 2.4.5. Functional Domains Annotation

Viral genomes were subjected to functional domain annotation using the online tool InterPro v.106.0 [29]. The output files were imported into Geneious Prime for visualization and editing. Additional editing was performed using Inkscape.

## 3. Results

### 3.1. Data Processing

Of all the collected samples, a total of 33 samples representing the 26 collected animals were eligible for sequencing. Approximately 1.26 billion reads were obtained, and about 607.6 million (~48.2%) were validated for genome assembly after data filtering (Table 1). From these, only 2,657,012 (~0.43% of filtered and ~0.21 of total reads) were classified as viral reads. Samples 17_MA7043 and 29_CH9914 were the ones with fewer and samples 26_RO22829 and 14_MA7042 the ones with more viral reads, respectively.

The morphological identification of each animal was confirmed by the analysis of the CytB subunit of their mtDNA (accession numbers at Table S1). The 26 animals consisted of 3 species of bats, 4 species of opossums and 5 species of rodents.

### 3.2. Viral Diversity

**Figure 2:**
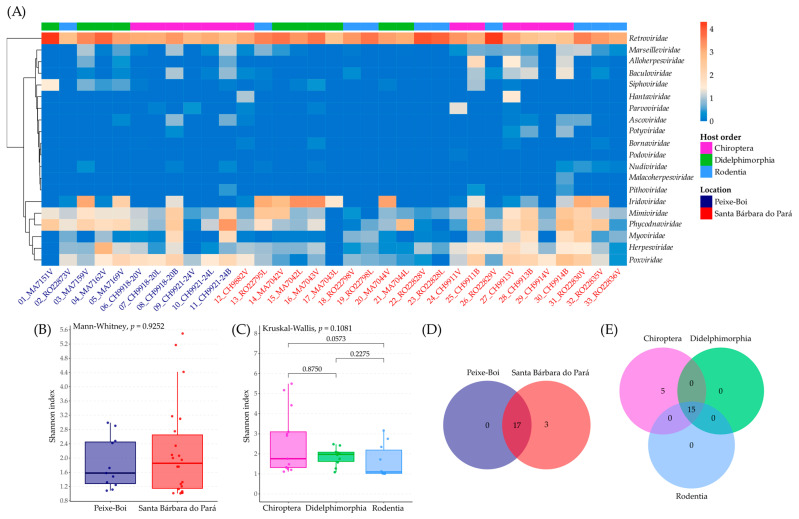
Viral Diversity analysis in small mammals from Peixe-Boi and Santa Bárbara do Pará. Heatmap representing the relative abundance (log-transformed scale) of viral families detected in different samples, grouped hierarchically by similarity of viral composition. The columns represent the samples, with the tissue type represented by letters V, L, and B and the location by colors blue or red, and the rows correspond to the viral families (**A**), boxplot comparing the Shannon diversity index between the two collection sites (**B**), boxplot of the Shannon diversity index comparing three host orders with adjusted *p*-values indicated for pairwise comparisons (**C**), Venn diagrams showing the number of shared and exclusive viral families between the two collection sites (**D**), and between the host orders (**E**).

The identified vOTUs from the two municipalities and three host orders comprised RNA and DNA viral families. Based on the dual alignment against the nr and RefSeq databases and the mapping against the filtered reads, they were assigned into 20 viral families, and the most abundant family was *Retroviridae*, which also was the only one identified in all samples. Then, *Phycodnaviridae*, *Iridoviridae*, *Herpesviridae*, and *Poxviridae* were also very abundant (Figure 2A, Table S2).

Considering alpha diversity, the Shannon index for location showed similar medians for both municipalities, which is corroborated by the Mann–Whitney test with *p* = 0.9252, and suggests that viral diversity is similar regardless of the location (Figure 2B, Table S3). The Shannon index for host order also presented close medians, with a *p* = 0.1081 by the Kruskal–Wallis test, suggesting the absence of significant differences in viral diversity (Figure 2C, Table S3). The Dunn test did not reveal any significant diversity differences between the host orders.

The richness analysis showed that both locations share 17 viral families and Santa Bárbara do Pará presented three exclusive viral families: *Hantaviridae*, *Malacoherpesviridae*, and *Podoviridae* (Figure 2D). However, their frequency among the samples and their abundance of reads was extremely limited (Table S3). The analysis for host orders showed that 15 viral families were shared and five are exclusive of bats: *Hantaviridae*, *Malacoherpesviridae*, *Pithoviridae*, *Podoviridae*, and *Potyviridae* (Figure 2E). Just like the other richness analysis, their frequency among the samples and their abundance of reads was very low (Table S3).

The beta diversity analysis was based on the Bray–Curtis dissimilarity matrix (Figure S1) and the first two components together explained 66.1% of the variation (45.8 + 20.3 = 66.1%). PCoA1 and PCoA2 coordinates for each sample are presented at Table S4. The beta diversity value (0.5669) revealed a high degree of variability in viral community composition among the 33 samples (Table S5). The PCoA analysis showed overlapping viral community composition in all comparisons: location vs. host order, tissue vs. host order, and season vs. location. The main viral families leading to the separation of viral communities were *Iridoviridae*, *Phycodnaviridae*, and *Retroviridae*.

**Figure 3:**
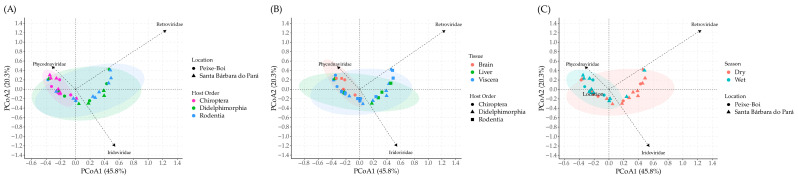
Beta diversity analysis of viral families considering location and host order (**A**), beta diversity analysis of viral families considering tissue and host order (**B**), and beta diversity analysis of viral families considering season and location (**C**).

Based on location vs. host order comparison, the host order had a strong influence according to the location (*p* = 0.005; R^2^ = 0.3143). The location (*p* = 0.568; R^2^ = 0.0213) itself had no significant effect on viral composition. However, when associated with host order, which itself had highly significant influence (*p* = 0.002; R^2^ = 0.2072), it strengthened the host order effect (Figure 3A).

The tissue type had a significant effect (*p* = 0.016; R^2^ = 0.15217), which was strongly influenced by the host order (*p* = 0.001; R^2^ = 0.40212) (Figure 3B). Both biological factors had the most significant effects on viral composition. Finally, the season also had a significant effect (*p* = 0.011; R^2^ = 0.12103), but no significant effect (*p* = 0.061; R^2^ = 0.15977) was observed when combined with location (Figure 3C).

### 3.3. Bat Endogenous Retroviruses (Retroviridae)

Bat endogenous retrovirus (ERV) contigs were detected in all bat samples. *Desmodus rotundus* endogenous retrovirus (DrERV) sequences from a *D. rotundus* bats from Pará were indicated as the best hits for the detected contigs. A sequence from Gurupá (Pará, OR344923) was used as a reference sequence. Considering the endogenous nature, it was decided to curate the sequences obtained from the viscera samples, which were the common sample type sequenced from all bats and with the higher alignment coverage with bat endogenous retroviruses.

Based on the cited reference, four bat endogenous sequences with the main retroviruses genes—gag, pro, pol, and env—were recovered from the 06_CH9918/20, 09_CH9921/24, 12_CH9882, and 27_CH9913 samples, all from *Carollia* bats. A sequence containing only the pol gene was recovered from the 24_CH9911 sample (*R. pumilio*). The LTR regions were not obtained for any of the sequences. Although contigs corresponding to the bat endogenous retrovirus were found, it was not possible to recover complete sequences for any of the genes in the 29_CH9914 (*C. perspicillata*) sample and its equivalent brain sample.

**Figure 4:**
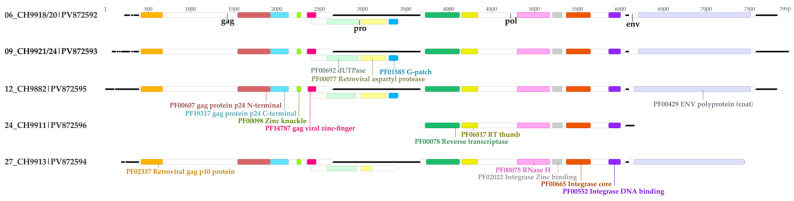
Functional domains in the obtained bat ERV sequences. Each domain is represented and named by color.

The four sequences with the main retrovirus genes had an average length of 7598 nt and the pol sequence from the 24_CH9911 sample had 2483 nt (Table 2). The gag gene had an average length of 2169 nt, pro had 1021 nt, pol had exactly 2388 nt in all sequences, and env had an average length of 1445 nt. None of the genes in the five sequences were predicted to produce protein truncating variants, and all functional domains common to retroviruses were identified (Figure 4), including throughout the length of the pol and env genes, which have several stop codons in DrERV sequences. The average nucleotide identity between the obtained sequences and the DrERV sequence from Gurupá was of 75.76% (Table 2) and the average amino acid identity for the gag, pro, pol, and env genes were of 64.50%, 72.97%, 91.97%, and 86.2%, respectively (Table S6).

**Figure 5:**
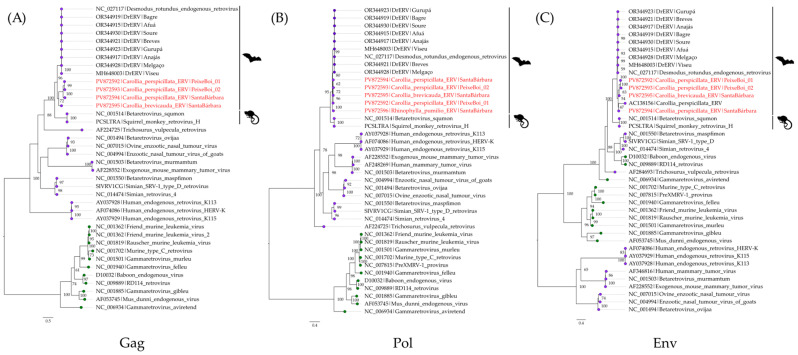
Maximum likelihood phylogenetic tree based on the nucleotide alignment of the gag (**A**), pol (**B**), and env (**C**) genes of retroviruses. The best nucleotide substitution models were the TVM + F + I + G4 for the gag and pol genes and the TVM + F + G4 for the env gene. Branches with lilac tips represent betaretroviruses and green tips represent gammaretroviruses. The obtained bat ERV sequences are highlighted in red.

The phylogenetic analysis was based on the nucleotide alignment of gag, pol, and env genes—as they are commonly utilized for retroviral phylogenetic analyses—from 32 retroviruses grouped in the *Betaretrovirus* and *Gammaretrovirus* genera along with the obtained bat ERV sequences. In all trees, the obtained bat sequences clustered into a sister clade to the DrERVs sequences. Together, they constitute a major bat ERV clade and are related to sequences from the *Betaretrovirus squmon* (Figure 5). Additionally, the obtained topologies are supported by phylogenetic signals of 65.9%, 62.1% and 68.5% for the gag, pol, and env genes, respectively (Figure S2).

### 3.4. Mobatvirus (Hantaviridae)

Contigs for the *Hantaviridae* family were detected in two viscera samples from bats from Santa Bárbara do Pará: 12_CH9882 (*C. brevicauda*) and 27_CH9913 (*C. perspicillata*), which were collected six months apart. In both samples, it was possible to recover contigs for each of the three segments of hantaviruses. The three sequences from the 12_CH9882 sample were already described at [30] and were characterized as being a strain of the Buritiense virus (BURV), which clusters within the *Mobatvirus* genus.

**Figure 6:**
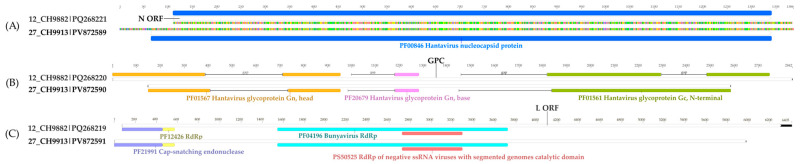
Functional domains in the S (**A**), M (**B**), and L (**C**) segments of the Buritiense virus. Each domain is represented and named by color.

From the 27_CH9913 sample, sequences for each segment were obtained. The recovered S segment (nucleoprotein) sequence is 1386 nt long, with an 8.7× genome coverage, and covering the complete ORF. The sequence presents the higher nucleotide and amino acid identity with the BURV S sequence from the 12_CH9882 sample, which are 99.13% and 99.75%, respectively (Table S7). In turn, the latter sequence is 113 nucleotides shorter and does not cover the entire nucleoprotein ORF. As for the functional domain, both share the hantavirus nucleocapsid protein domain (Figure 6A).

A portion of the M segment (glycoprotein precursor, GPC), with 2437 nt was obtained, with a 2.5× genome coverage, and presents three gaps based on the reference sequence (NC_055634, *Mobatvirus robinaense*). Although there was a BURV M sequence described from the 12_CH9882 sample, it had just 690 nt. The new sequence for the segment was used as a reference to revise the original sequence through a map to reference alignment of the 12_CH9882 sample reads. The sequence was extended to 2842 nt, considering four gaps (Figure 6B). Both share 96.45% and 95.82% of nucleotide and amino acid identity, respectively (Table S7). They also share the same glycoprotein domains (Figure 6B).

Regarding the L segment (large protein), the obtained sequence is 5979 nt long, with a 4.2× genome coverage. It shares greater nucleotide identity (92.8%) with the BURV L sequence from the 12_CH9882 sample, with whom it also shares a 94.68% amino acid identity (Table S7). Both sequences had the same four functional domains annotated, which are related to viral replication (Figure 6C).

**Figure 7:**
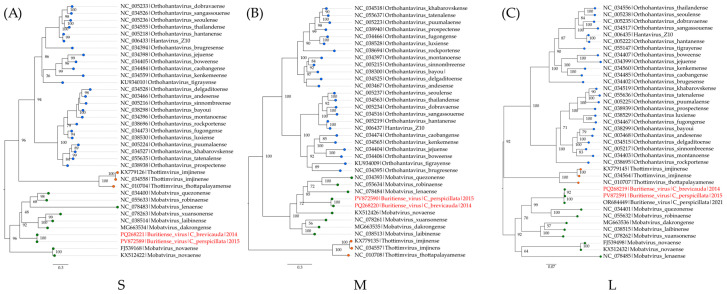
Maximum likelihood phylogenetic tree based on the amino acid alignment of the S (**A**), M (**B**), and L (**C**) segments of hantaviruses. The best amino acid substitution models were the Q.insect + I + G4 for the S and M segments and the Q.insect + I + R5 for the L segment. Branches with blue tips represent orthohantaviruses, orange tips represent thottimviruses, and green tips represent mobatviruses. The obtained BURV sequences are highlighted in red.

The phylogenetic analysis for the three segments considered amino acid sequences for each segment of 35 hantaviruses along with the obtained BURV sequences. The tree topologies are supported by high internal anchoring values and the BURV sequences clustered together within the *Mobatvirus* clade in each tree, but with different internal positioning (Figure 7). Additionally, the obtained topologies are supported by phylogenetic signals of 91.6%, 94.4%, and 99.6% for the S, M, and L segments, respectively (Figure S3).

## 4. Discussion

Deforestation in the Amazon region is leading to natural habitat loss and directly affects the dynamics of its biodiversity, forcing wild animals to adapt to degenerated ecosystems and to move closer to humans, livestock, and other domestic animals. These circumstances are conducive to the emergence of pathogens, highlighting the need for One Health research studies [31,32].

The virome analysis of wild animals is a useful tool for One Health, as it sheds light on their viral diversity. It can be crucial to surveillance preparedness, given the possibility of identifying potential threats to public health [33]. It is estimated that 40,000 viruses have mammals as reservoirs, of which about 10,000 have zoonotic potential [34]. Bats and rodents are reservoir hosts of a high number of zoonotic viruses and, although less studied, opossums have also been acknowledged as hosts for some viruses, most of which are shared with humans [35].

In this study, we analyzed 33 samples from 26 collected animals. Of the 1.26 billion generated reads, only 2.65 million (~0.21%) were identified as viral. Low coverage of viral genomes is quite common in virome studies, such as some that also focus on wildlife in the Amazon region [36,37,38,39].

The viral families with higher abundance were *Retroviridae*—present in all samples—*Phycodnaviridae*, and *Iridoviridae*. Given that tissue samples were analyzed in this study, reads associated with the *Retroviridae* family must be endogenous elements integrated into the host genome, as the recovered bat ERVs. The *Phycodnaviridae* and *Iridoviridae* families comprise giant viruses from algae and ectothermic animals, respectively. The detection of these viral families in mammalian tissue samples is unlikely to reflect active infections, but rather may result from feeding habits, environmental contamination, or limitations in the current pipeline [37,40,41]. In contrast, the detection of families such as *Herpesviridae* and *Poxviridae* are well-documented mammalian pathogens [42], and their detection could potentially reflect active infections. However, given the metagenomic approach employed in this study, our data cannot conclusively distinguish between transient and replication-competent components of the virome. These results from both RNA and DNA viruses highlight the importance of cautious interpretation of metagenomic data and emphasize the need for complementary approaches, such as PCR, viral isolation, or histopathological analyses to confirm whether they are truly infecting mammalian hosts [42,43].

Considering the alpha diversity, there were no statistically significant differences in viral diversity by location or host order. Despite the lack of significance, the Shannon index and the richness analysis revealed that the Chiroptera order and the Santa Bárbara do Pará municipality showed a tendency toward more variability, possibly associated with the high ecological plasticity and mobility of bats, which can explore multiple habitats, interact with a variety of prey and plant resources [44]. Also, a higher number of samples were analyzed from this location. From the exclusive families in both richness analysis, only the *Hantaviridae* family was well represented in terms of abundance, which is related to the BURV detection in two bat samples from Santa Bárbara do Pará.

The beta diversity analysis suggested that biological and seasonal factors had more influence on the composition of viromes than geographic variation. It makes sense considering that both localities are only 134 km apart and are covered by the same vegetation physiognomy, which reduces environmental heterogeneity. There was a clear separation of samples based especially on host order and tissue type, while their interaction had an even more significant effect on viral composition, which may be related to the specificity of host taxonomy and coevolutive adaptations of tropism [45]. In addition, the absence of location influence in viral composition was also observed in a virome study of wild rodents from two neighboring localities in southeastern Pará State [37]. Together, these findings reveal that the organization of viral communities is multifactorial, being modulated by interactions between host factors, properties of the viral families and environmental conditions.

The lifecycle of retroviruses includes the reverse transcription of genomic RNA into DNA, which integrates into the host genome as a provirus. If the infection affects germline cells, the provirus can be transferred to descendants. These inherited genetic elements that have survived natural selection following exogenous retrovirus infection in an ancestor of the species are known as ERVs [46,47]. It is estimated that sequences derived from ERVs constitute about 8% of the human genome and mammalian ERVs appear to have been formed constantly throughout mammalian evolution over millions of years. Although most EVRs appear to be defective, some have been identified as functional in mice [47]. Furthermore, ERVs can generate virus-like particles. More than 50 species of bats have been reported with ERVs, and the first one was described in 2004 from a *C. perspicillata* genome, the *Carollia perspicillata* ERV betaretrovirus 5 (CpERV-*β*5). Genetically related to betaretroviruses, the CpERV-*β*5 is an incomplete and defective ERV [48].

Five bat ERVs sequences were recovered from viscera samples from three bat species. All *Carollia* ERV sequences comprise the four main retrovirus genes and the obtained *R. pumilio* ERV (RpERV) comprises only the pol gene, where its length was the same in sequences from all species. Furthermore, it is the gene with the highest nucleotide and amino acid identity between them and the reference sequence and among themselves, which may be evidence that it is a well conserved gene.

Interestingly, the genes from all sequences do not present deleterious mutations like premature stop codons, as commonly observed in ERVs. As a comparison, the CpERV-*β*5 has a complete deletion of pol and pro genes and DrERVs have protein truncated variants of pol and env genes [49,50]. On the other hand, intact genes were observed in a *Myotis lucifugus* ERV [51]. It was possible to recover functional domains common to retroviruses in all genes, even throughout the pol and env genes. In addition, all sequences clustered in a major bat ERV clade related to the *Betaretrovirus squmon* in all trees, an already described clustering pattern [49,50].

Although these and more samples from *Carollia* bats need to be studied in terms of viral isolation and titration in several cell lines, molecular clock and LTR analysis to exclude the possibility of being exogenous retroviruses, it is quite possible that the obtained sequences from Amazonian *Carollia* bats could represent ERVs that were recently endogenized or are undergoing endogenization, as previously observed in South Korean *Rhinolophus ferrumequinum* bat population [52]. Additionally, when an ERV is detected in some, but not all individual hosts from the same species and location, it has not been fixed yet or is still under endogenization [48], even though the absence of ERVs in both CH9914 samples could also be a result of a lower sequencing coverage.

Originally established as a genus of the former *Bunyaviridae* family and thought to comprise only rodent-borne viruses, hantaviruses were re-organized as a family in 2017, and its known host range was widely expanded, now comprising fish, reptiles, moles, shrews, and bats [53]. The *Mobatvirus* genus was created in 2018 and includes seven species described from shrews, moles, and bats. Their S segment ranges from 1.3 to 2.0 kb, the M segment from 3.4 to 3.9 kb, and the L segment from 6.4 to 6.6 kb. Bat-borne mobatviruses recognized by the ICTV were discovered in Emballonuridae, Hipposideridae, and Pteropodidae bats from Southeast Asia [54].

The BURV was firstly reported from a *C. perspicillata* collected in Timon, Maranhão State, Brazil in 2021. Its partial L segment was recovered from a viscera sample containing fragments of the spleen, lymph nodes, heart, and lungs and clustered within the *Mobatvirus* clade [55]. Subsequently, another BURV strain was described from 12_CH9882 sample of this study, becoming the first description of BURV in the Amazon region [30].

From a viscera sample of a *C. perspicillata* (27_CH9913), another BURV strain has now been obtained, also from Santa Bárbara do Pará, but from 2015. The novel M sequence was pivotal to revise the M sequence from the previously described sample. The recovered coding-complete S sequence is 1386 nt long which is within the range of mobatviruses S segment length. The three new sequences clustered together within the *Mobatvirus* clade with the same clustering pattern observed in previous studies [30,55].

Interestingly, other BURV strains were recently described in several *C. perspicillata* collected in Darién Province, Panamá, in 2023. Partial sequences for segments S (two), M (one), and L (four) were recovered from lung samples and clustered with Brazilian strains [56]. These data corroborate BURV circulation in *Carollia* bats in Central and South America [30,55,56]. Considering that lung fragments also compose the viscera samples from Timon and Santa Bárbara do Pará, it is possible that it could potentially be the primary tropism site of the virus. Even though only viruses from the *Orthohantavirus* genus—classical rodent-borne hantaviruses—are known to cause disease in humans, the World Health Organization categorizes the entire *Hantaviridae* family as of high priority to public health emergency preparedness [57], which underscores the importance of surveillance and complete characterization of BURV, as well as predicting its zoonotic potential, since both *Carollia* species are widespread throughout Brazil and are commonly found in disturbed areas [58,59].

## 5. Conclusions

Considering the high biodiversity and intense interaction between species in the Amazon region, the vulnerability scenario resulting from anthropization demonstrates the need for One Health surveillance to anticipate potential threats to public health. This study analyzed the virome of eleven bats, seven opossums, and eight rodents from two municipalities in the Pará State. The analysis suggested that host order, tissue type, and season have influence on the composition of viromes, while location had no significant influence, which means that organization of viral communities is multifactorial, reinforcing the need for continuous surveillance in wild animals, especially in the Amazon region.

In addition, it was possible to recover five viral genomes of bat ERVs with preserved genome architecture and genes, which could be indicative of a recent endogenization phenomenon or an ongoing process of endogenization. Another BURV strain was also recovered, supporting its circulation in Santa Bárbara do Pará and expanding the knowledge on its S and M segments. Both findings need to be further investigated using complementary techniques to fully characterize the viruses. Specifically for BURV, it is also necessary to better understand its distribution and zoonotic potential.

## Figures and Tables

**Table 1:** Sample and data processing information.

Location	Sample ID	Tissue	Host Species	Collection Date	Total Reads	Fastp	SortMeRNA	Viral Reads
Peixe-Boi (Ananin village)	01_MA7151	Viscera	*Cryptonanus agricolai*	11 September 2015	52,309,212	47,490,186	8,498,016	20,423
	02_RO22873	Viscera	*Rattus rattus*	12 September 2015	49,198,384	45,713,586	12,068,688	6731
	03_MA7159	Viscera	*Marmosops pinheiroi*	14 September 2015	52,770,248	47,445,710	16,385,644	65,091
	04_MA7162	Viscera	*Metachirus myosuros*	15 September 2015	49,699,504	46,350,714	13,363,622	198,399
	05_MA7169	Viscera	*Philander opossum*	18 September 2015	57,013,870	52,138,166	14,284,912	25,126
	06_CH9918/20	Viscera	*Carollia perspicillata*	25 May 2016	46,011,536	42,632,696	26,247,326	30,878
	07_CH9918/20	Liver			34,922,698	31,903,538	19,319,456	20,866
	08_CH9918/20	Brain			42,923,108	39,645,450	6,098,404	14,962
	09_CH9921/24	Viscera	*Carollia perspicillata*	26 May 2016	10,162,764	10,161,884	9,438,986	7148
	10_CH9921/24	Liver			43,566,608	40,290,140	16,003,212	18,795
	11_CH9921/24	Brain			33,354,502	30,545,230	11,003,166	29,856
Santa Bárbara do Pará (Expedito Ribeiro settlement)	12_CH9882	Viscera	*Carollia brevicauda*	17 October 2014	60,996,864	55,590,760	38,036,772	47,276
	13_RO22795	Liver	*Echimys chrysurus*	20 October 2014	72,620,606	68,639,944	62,188,324	290,584
	14_MA7042	Viscera	*Marmosops pinheiroi*	23 October 2014	60,191,660	56,574,576	54,504,442	323,359
	15_MA7042	Liver			27,118,572	25,941,438	21,622,680	92,321
	16_MA7043	Viscera	*Philander opossum*	23 October 2014	41,365,578	37,067,938	36,728,954	238,408
	17_MA7043	Liver			14,035,228	13,518,008	478,446	230
	18_RO22798	Viscera	*Oecomys paricola*	24 October 2014	48,997,760	41,275,094	27,560,462	50,691
	19_RO22798	Liver			10,378,774	9,977,804	3,811,070	16,257
	20_MA7044	Viscera	*Marmosops pinheiroi*	25 October 2014	45,637,316	40,316,828	32,671,306	94,728
	21_MA7044	Liver			28,369,886	25,422,456	16,867,168	28,167
	22_RO22828	Viscera	*Oecomys paricola*	28 October 2014	31,158,412	29,536,146	26,805,502	260,923
	23_RO22828	Liver			17,589,744	16,813,682	11,660,122	83,672
	24_CH9911	Viscera	*Rhinophylla pumilio*	07 April 2015	43,168,902	39,104,674	21,264,060	44,178
	25_CH9911	Brain			33,583,454	30,474,710	4,988,994	9857
	26_RO22829	Viscera	*Nectomys rattus*	08 April 2015	36,475,646	32,978,740	27,859,838	508,107
	27_CH9913	Viscera	*Carollia perspicillata*	09 April 2015	30,715,366	28,149,712	9,439,310	15,579
	28_CH9913	Brain			35,577,070	32,750,072	9,624,060	10,227
	29_CH9914	Viscera	*Carollia perspicillata*	09 April 2015	34,483,028	31,526,694	5,905,172	2030
	30_CH9914	Brain			24,492,902	22,588,498	10,498,444	20,738
	31_RO22830	Viscera	*Guerlinguetus aestuans*	08 April 2015	39,958,576	36,804,108	5,893,486	28,790
	32_RO22835	Viscera	*Rattus rattus*	16 April 2015	21,722,476	18,281,404	14,685,132	40,663
	33_RO22836	Viscera	*Oecomys paricola*	16 April 2015	31,059,340	27,370,724	11,802,244	11,952
	Total		*-*	-	1,261,629,594	1,155,021,310	607,607,420	2,657,012

**Table 2:** Information about the five obtained bat ERV sequences.

Origin Sample	Host Species	Length (nt)	Coverage (×)	Nt Identity (%) with Reference
06_CH9918/20	*C. perspicillata*	7473	26.5	74.08
09_CH9921/24	*C. perspicillata*	7748	15.4	71.99
12_CH9882	*C. brevicauda*	7805	54.2	75.36
24_CH9911 ^1^	*R. pumilio*	2483	82.6	69.3
27_CH9913	*C. perspicillata*	7097	9.4	88.11

^1^ Only the pol gene sequence was recovered.

## Data Availability

The study sequences were deposited in GenBank under accession numbers PV872589 to PV872596, and the raw sequencing data have the following SRA numbers: SRR30438315, SRR33632182, SRR34564527 to SRR34564529, and SRR35058925 to SRR35058952. The CytB sequences were deposited under accession numbers PQ276609, PV684749, and PX207651 to PX207681.

## Supplementary Materials

The following supporting information can be downloaded at https://www.mdpi.com/article/10.3390/v17091251/s1. Figure S1: Bray–Curtis dissimilarity matrix; Figure S2: Phylogenetic signal mapping diagrams showing the quartet analysis for the gag (A), pol (B), and env (C) genes alignments; Figure S3: Phylogenetic signal mapping diagrams showing the quartet analysis for the S (A), M (B), and L (C) segment alignments; Table S1: Results of CytB analysis, as well as CytB and SRA accession numbers; Table S2: Viral read classification by viral family and number of reads; Table S3: Richness and Shannon diversity index metric analysis; Table S4: PCoA1 and PCoA2 coordinates for each sample.; Table S5: Beta-diversity metrics; Table S6: Identity matrices for main retroviruses genes; Table S7: Identity matrices for each hantavirus segment.
