# Supplementary material for: Virome Analysis of Small Mammals from the Brazilian Amazon
Source: Viruses. 2025 Sep 16;17(9):1251. doi: 10.3390/v17091251 (PMC12474034; doi:10.3390/v17091251)
Supplement: Supplementary file 1 [file viruses-17-01251-s001.zip › Supplementary Figures.pdf]

Supplementary Figures

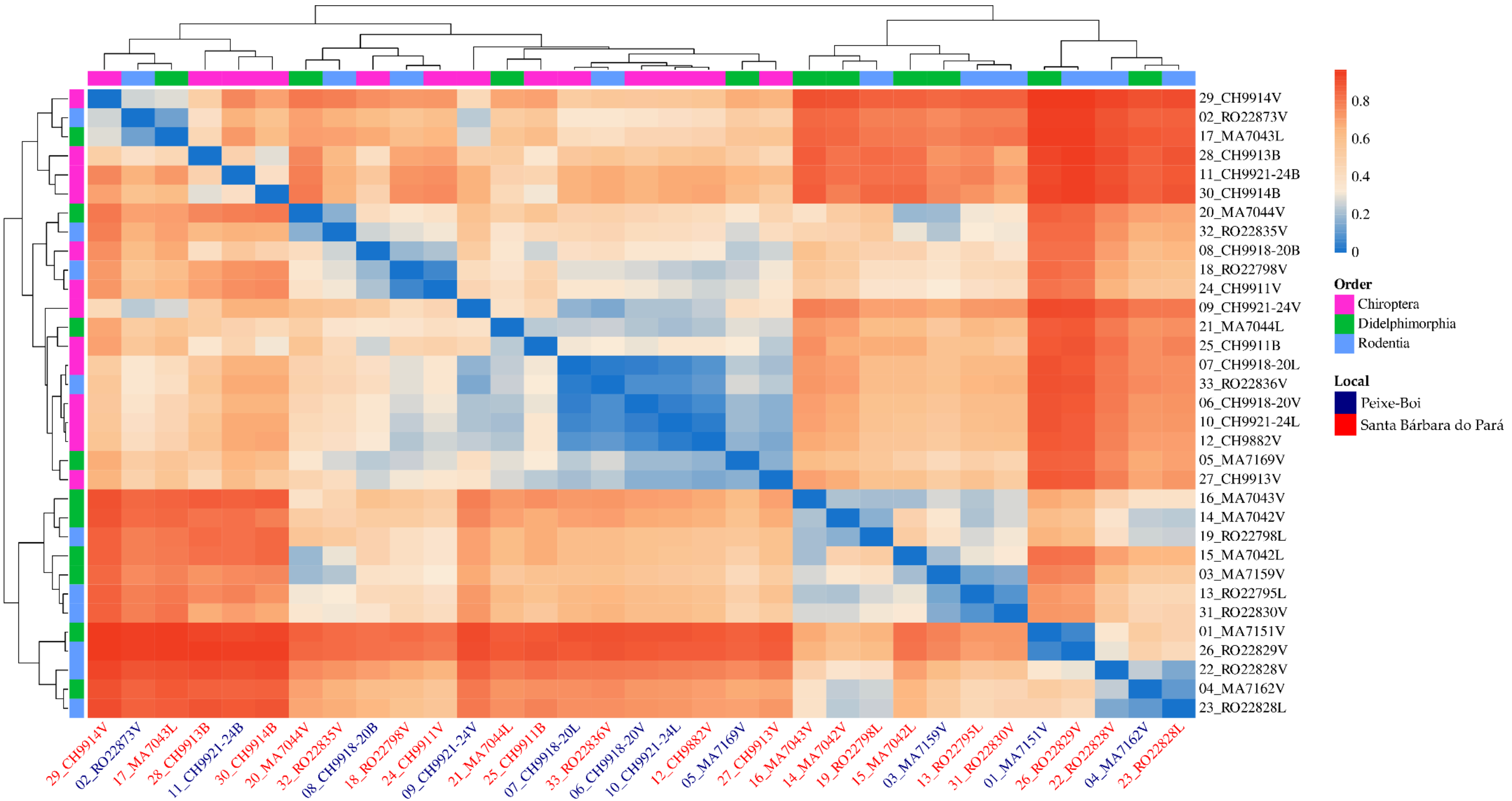

Figure S1. Bray–Curtis dissimilarity matrix.

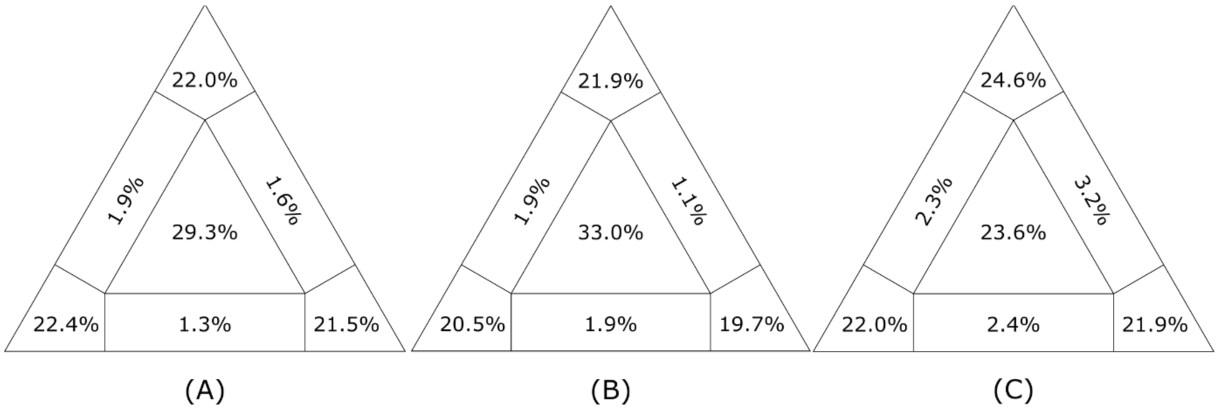

Figure S2. Phylogenetic signal mapping diagrams showing the quartet analysis for the gag (**A**), pol (**B**), and env (**C**) genes alignments.

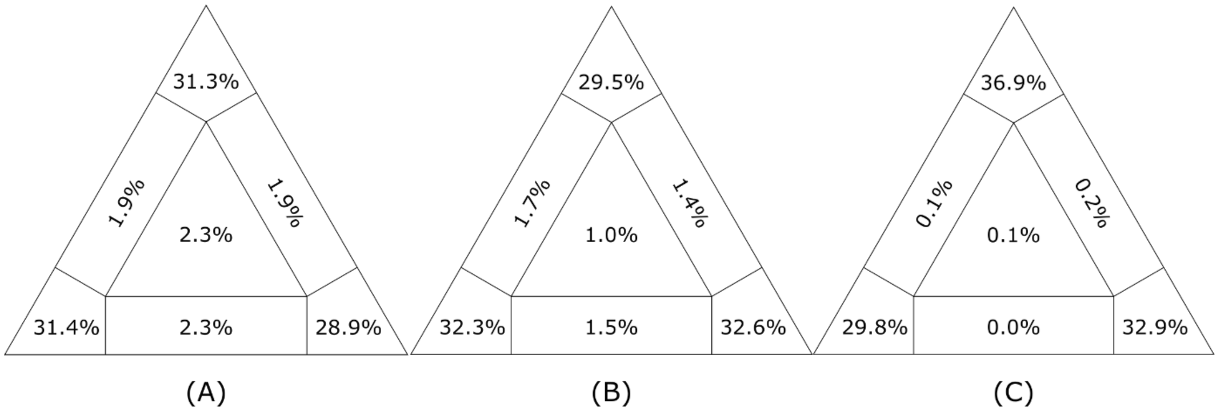

Figure S3. Phylogenetic signal mapping diagrams showing the quartet analysis for the S **(A)**, M **(B)**, and L **(C)** segment alignments.
